# Ecological stoichiometric characteristics of the straw-soil-microbial system in the straw checkerboard barriers across different laying years

**DOI:** 10.7717/peerj.21569

**Published:** 2026-07-30

**Authors:** Jieru Kong, Bingru Liu

**Affiliations:** 1 School of Bioscience and Bioengineering, North Minzu University, Yinchuan, China; 2 Ningxia Baijitan National Field Observation and Research Station of Forest Ecosystem, Lingwu, China; 3 State Key Laboratory of Ecological Protection of Agro-pastoral Ecotones in the Yellow River Basin, National Ethnic Affairs Commission of China, Yinchuan, China

**Keywords:** Laying years of straw checkerboard barriers, Straw-soil-microbial, C, N, and P elements, Ecological stoichiometry, Path analysis

## Abstract

This study utilized ecological stoichiometry to analyze the changes in the carbon (C), nitrogen (N), and phosphorus (P) within the straw-soil-microbial system across different laying years of straw checkerboard barriers in an arid sandy area, providing a reference for understanding the cycling process and regulation mechanism of these nutrients in such an environment. We selected straw checkerboard barrier areas laid for 1, 2, 5, 10, and 24 years as research plots with bare sandy land as the control. We collected soil samples at 0–20 cm and 20–30 cm depths along with straw samples of different degradation levels to investigate the changes in C, N, and P contents in the straw-soil-microbial system. The results showed that as the laying years of straw checkerboard barriers increased, straw N content, straw C:P and N:P ratios increased, while straw C content, straw C:N ratio decreased. Meanwhile, straw P content initially increased and then declined. The laying years of straw checkerboard barriers significantly impacted the contents of C, N, and P and the stoichiometry of straw. The contents of C, N, and P in soil and soil microbial biomass (MB) at different depths showed different responses, and their stoichiometry also varied based on the laying years of straw checkerboard barriers. Straw C and N contents showed a direct and significant positive impact on soil C-N-P stoichiometry, while straw N content displayed a direct and significant negative influence on C-N-P stoichiometry of soil MB. The primary factors influencing the changes in C, N, and P contents of the straw were soil total carbon (TC) content, MBN:MBP, and C:P ratios. The regulation of C-N-P cycling by the laying years of straw checkerboard barriers is a complex system involving “multiple components, multiple processes, and multiple scales”. Further research in this area should focus on mechanism analysis and scenario expansion.

## Introduction

Desertification not only endangers human habitation but also impacts sustainable resource development, the environment, and the social economy (Reynolds et al., 2007; Guo et al., 2015). Desertification has been accelerating due to climate change and disturbances by human activities leading to the inhibition of the natural restoration capacity of the ecosystem (Zhang & Deng, 2020). A variety of sand management technologies including mechanical and biological sand fixation technology have been implemented to prevent the aggravation of land desertification (Xu et al., 2020). Mechanical sand control technology is required to achieve the effects of wind prevention and sand fixation because biological sand fixation technology cannot be directly implemented due to limitations in water sources and climatic conditions (Liu et al., 2011). Mechanical sand fixation technology utilizes materials like biomass (mainly crop straw) and minerals (mainly clay and gravel) to modify the surface roughness and structure of near-surface wind-sand flow by creating barriers on the sand surface (Yuan et al., 2012; Chen et al., 2014). The straw checkerboard barriers have emerged as a relatively common wind prevention and sand fixation method in mechanical sand fixation technology due to its advantages of being environmentally friendly, minimal technical requirements for construction, and ease of operation (Qu et al., 2006).

The straw checkerboard barriers are made by arranging wheat straw, rice straw, and reeds in square grids on the mobile sand dunes (Yang, 2023). Naturally, straw checkerboard barriers will decay and decompose over time due to a variety of environmental factors as they are made up of plant straw. When the decomposed plant materials mix with the sand, they serve a variety of roles including conserving water sources, improving soil quality, promoting reproduction and growth of microorganisms in the shifting sand, and creating a favorable habitat for pioneer plant colonization (Ma et al., 2024; Jiang, 2020; Yang et al., 2024). Studies have shown that straw carbon (C), nitrogen (N), and phosphorus (P) elements will gradually be transformed into forms such as humus, ammonium nitrogen and inorganic phosphates under the decomposition action of microorganisms, and be available for plants to absorb and utilize (Mao et al., 2022). The main source of nutrients for plants and microorganisms in terrestrial ecosystems is soil, which serves as both the site conditions for plants and the living environment for microorganisms (Sheng, Liu & Xiong, 2013; Zhou et al., 2023). Soil microorganisms are important decomposers and play a significant bridging role between plants and soil (Tharanath, Upendra & Rajendra, 2024). C, N, and P are the most fundamental components of an ecosystem. The stoichiometric characteristics and coupling relationships of C, P, and N in plants, soil, and microorganisms can, to some degree, regulate the material circulation and energy flow of ecosystems (Yu et al., 2013). The ecological stoichiometric ratio of plant straw regulates the microbial utilization efficiency of straw substrates, community structure and functional activity, thereby mediating the improvement of soil physicochemical properties and nutrient cycling processes.

Currently, a lot of research has been conducted on topics such as the machinery used to construct straw checkerboard barriers and the ecological impact of straw checkerboard barriers on afforestation. However, there is a lack of systematic research on the ecological stoichiometric characteristics of C, N, and P in straw-soil-microbial systems during laying years of straw checkerboard barriers. Hence, this study used straw checkerboard barriers with laying years of 1, 2, 5, 10, and 24 as research plots, and bare sandy land as the control, based on the theory of ecological stoichiometry.

The aim was to explore the changing patterns of C, N, and P contents and stoichiometric ratios in plant straw, soil, and microorganisms across different laying years of straw checkerboard barriers. The goal was to reveal the dynamic balance law and maintenance mechanism of C, N, and P within the ecosystem of straw checkerboard barrier areas. The findings of this study would provide a scientific basis for vegetation restoration, afforestation with straw checkerboard barriers, soil management, and sand control in arid and sandy regions.

## Materials and Methods

### Site description

This study was conducted in the wind-sand region of the Baijitan National Nature Reserve in Lingwu, Ningxia, located at the southwest edge of the Maowusu desert (106°20′22″∼106°37′19″E, 37°49′05″∼38°20′54″N). The area was dominated by *Caragana korshinskii*, *Hedysarum scoparium*, *Calligonum mongolicum*, and other desert plants. The southern section of the studied area bordered the Loess Plateau and was characterized by a hilly landscape with an elevation between 1,150 and 1,650 m. Meanwhile, the northern side was primarily dominated by mountainous deserts, with an average elevation of 1,250 m. The reserve featured a concentrated distribution of desert ecosystems such as arid sandy land, desert steppe, and mobile sand dunes. This reserve had a typical continental monsoon climate, with an average annual precipitation of 192.9 mm. Moreover, the reserve has four distinct seasons *i.e.,* late springs and early autumns, dry weather with little and concentrated rainfall, strong evaporation, large temperature differences, and long hours of sunshine. The predominant soil types are mainly sierozem and aeolian sandy soil.

### Sample collection

**Figure 1:**
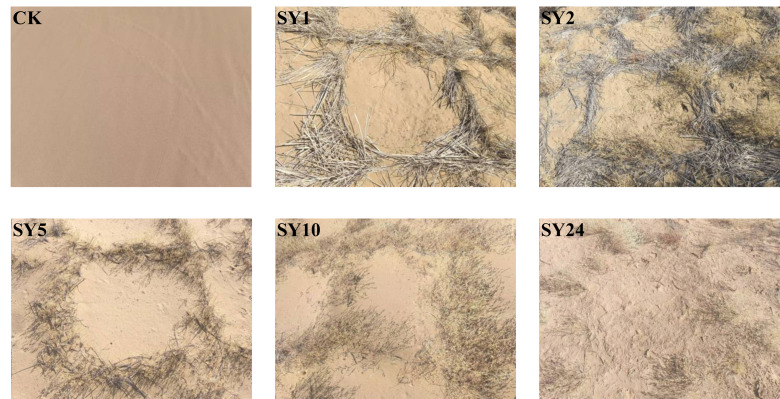
The basic situation of the research area.

In August 2023, research plots in the straw checkerboard barriers areas with similar slopes, the same aspects, and rice straw as the barrier material were selected for 1 (SY1), 2 (SY2), 5 (SY5), 10 (SY10), and 24 (SY24) years. A bare sandy area (CK) without straw checkerboard barriers was selected as the control. A total of six plots, each 100 m  ×  100 m in size and spaced at least 1,000 m apart, were used to collect samples (Fig. 1). Five 5 m   ×  5 m quadrats separated at least 20 m apart were randomly assigned as replicates within each sample plot. Soil samples were collected from 0–20 cm and 20–30 cm soil layers inside each quadrat using a micro-soil drill by following the five-point sampling approach. Moreover, rice straws with different levels of decomposition inserted into the soil were also collected.

The soil samples collected from the five different points were thoroughly mixed to form one composite sample. Each mixed soil sample was divided into two parts to determine different soil parameters. One part was allowed to air dry naturally and was preserved for the analysis of the contents of soil total carbon (Soil TC), total phosphorus (Soil TP), and total nitrogen (Soil TN). While, the other part was ground through a two mm sieve and kept in a refrigerator at 4 °C to determine soil microbial biomass (MB) C (Soil MBC), N (Soil MBN), and P (Soil MBP). Additionally, rice straw samples with different decomposition levels were dried in an oven at 65 °C, ground, and stored for the analysis of the total C (Straw C), total N (Straw N), and total P (Straw P) contents in the straw.

### Analysis of C, N , and P in straw, soil, and soil microbial biomass

Total C in straw and soil was measured using the combustion-non-aqueous titration method, while total N was determined *via* the H_2_SO_4_-H_2_O_2_ digestion-Kjeldahl method. Total P in straw and soil was analyzed using the HClO_4_-H_2_SO_4_ digestion-molybdenum blue colorimetric method (Bao, 2000). The contents of MBC, MBP, and MBN in the soil were assessed using the chloroform fumigation-extraction method (Guo, 2009). The straw-soil-microbial stoichiometry was calculated after calculating the C, N, and P contents.

### Statistical analysis

Data were compiled using Excel (2021) and analyzed using IBM SPSS Statistics v19.0 (IBM, Chicago, IL, USA) software. One-way analysis of variance (ANOVA) and Student’s test were used to compare the variations in C, N, and P contents and stoichiometric ratios of straw, soil, and MB across different and same laying years of straw checkerboard barriers. The relationship between laying years of straw checkerboard barriers and C, N, and P contents and stoichiometric ratios of straw, soil, and MB was analyzed using regression analysis. Spearman correlation heat map, Monte Carlo test, redundancy analysis (RDA), and Partial Least Squares Structural Equation Model (PLS-SEM) were used to examine the correlations among C, N, and P contents and stoichiometric ratios of straw, soil, and MB across different laying years of straw checkerboard barriers. PLS-SEM employs the plspm package in the RStudio to conduct partial least squares path analysis on the data. The exogenous variables include laying years and soil depth, while the endogenous variables are soil and microbial properties, Straw C, N, and P, as well as stoichiometries of C-N-P in straw, soil, and soil microbial biomass.

## Results

### Changes in straw contents of C, P, and N and stoichiometric ratios in straw checkerboard barriers across different laying years

We recorded variations in C, N, and P contents and stoichiometric ratios in straw within straw checkerboard barriers across different laying years (Table 1, Table S1). The straw C content exhibited a downward trend in an order of CK>SY1>SY2>SY5>SY10>SY24 as the laying years of straw checkerboard barriers increased. The straw C content of SY5, SY10, and SY24 was significantly lower compared to CK, SY1, and SY2 (*P* < 0.05). The straw N content showed an increasing trend as the laying years of straw checkerboard barriers increased. We observed significantly higher N content in SY10 and SY24 compared to CK, SY1, SY2, and SY5. Moreover, the straw N content of SY2 and SY5 was significantly higher than in CK (*P* < 0.05). As the laying years of straw checkerboard barriers increased, straw P content initially increased and subsequently decreased, with SY1 showing the highest P content. The straw C:N ratio decreased as the laying years of straw checkerboard barriers increased with CK showing a significantly higher C:N ratio than in SY1, SY2, SY5, SY10, and SY24 (*P* < 0.05). On the other hand, the straw C:P and N:P ratios increased as laying years of straw checkerboard barriers increased with SY24 displaying significantly higher C:P and N:P ratios compared to CK, SY1, SY2, SY5, and SY10 (*P* < 0.05).

### Changes in soil TC, TP, and TN contents and stoichiometric ratios in straw checkerboard barriers across different laying years

**Figure 2:**
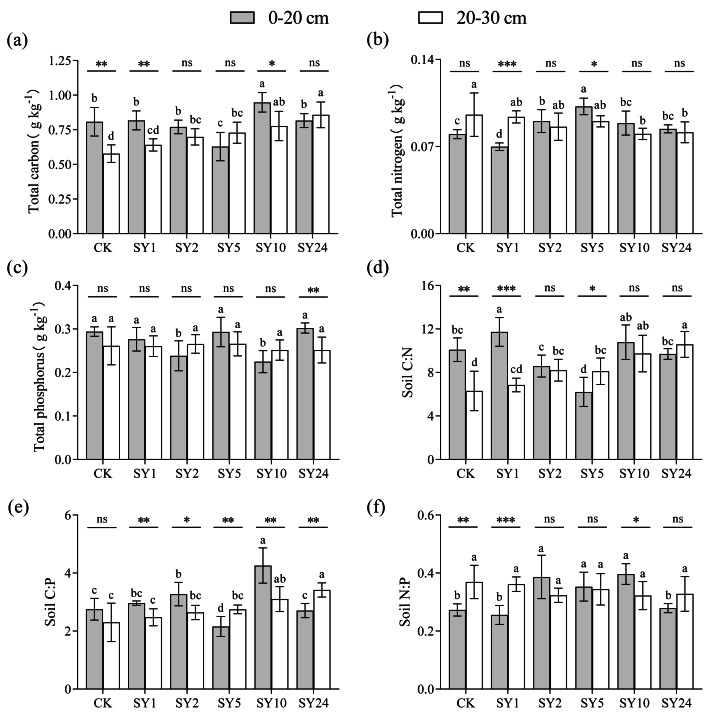
(A–F) Changes in soil contents of TC, TP, and TN and stoichiometric ratios. CK represents a bare sandy area without straw checkerboard barriers, while SY1, SY2, SY5, SY10, and SY24 represent straw checkerboard barriers of different laying years. Lowercase letters (such as a, b, c, d, ab, bc, and cd) denote significant differences between laying years of straw checkerboard barriers (*P* < 0.05). Significant variations (such as *** , ** , * , ns) between different soil layers (0–20 cm, 20–30 cm) of straw checkerboard barriers with the same laying year are indicated by asterisks *** (*P* < 0.001), ** (*P* < 0.01), * (*P* < 0.05), ns (no significant difference). Values are mean s ± S D (*n* = 5).

The comparison of TC, TN, TP contents, and stoichiometric ratios in soil across different laying years of straw checkerboard barriers revealed no obvious regular variations in soil TC, TN, TP, C:N ratio, C:P ratio, and N:P ratio in the 0–20 cm soil layer as the laying years increased (Fig. 2). The soil TC content, C:N ratio, and C:P ratio exhibited an increasing tendency in an order of CK<SY1<SY2<SY5<SY10<SY24 in the 20–30 cm soil layer as the laying years of straw checkerboard barriers increased. Additionally, soil TC content, C:N ratio, and C:P ratio in SY10 and SY24 were significantly higher than in CK and SY1 (*P* < 0.05). The soil TP content initially increased and then decreased with SY5 exhibiting higher TP contents as the laying years of straw checkerboard barriers increased. Soil TN content and N:P ratio did not show an obvious regular variations across different laying years of straw checkerboard barriers.

**Table 1:** The changes in straw C, P, N contents and stoichiometric ratios in straw checkerboard barriers across different laying years (mean ± SE). CK represents bare sandy area without straw checkerboard barriers, while SY1, SY2, SY5, SY10 and SY24 represent straw checkerboard barriers of different laying years. Different lowercase letters indicate significant differences between different laying years of straw checkerboard barriers (*P* < 0.05).

Research plots	Straw carbon (g kg^−1^)	Straw nitrogen (g kg^−1^)	Straw phosphorus (g kg^−1^)	Straw C:N	Straw C:P	Straw N:P
CK	350.47 ± 18.22a	3.71 ± 0.14c	5.36 ± 1.35a	94.64 ± 4.70a	51.70 ± 1.78d	0.55 ± 0.02d
SY1	348.42 ± 8.81a	4.22 ± 0.19bc	6.43 ± 0.34a	82.00 ± 3.97b	53.98 ± 2.80d	0.66 ± 0.04d
SY2	347.70 ± 12.68a	4.33 ± 0.17b	3.15 ± 0.15b	80.62 ± 3.30b	111.76 ± 7.40c	1.39 ± 0.08c
SY5	304.42 ± 8.48b	4.43 ± 0.14b	2.98 ± 0.43b	78.05 ± 3.69b	109.59 ± 13.07c	1.40 ± 0.16c
SY10	264.53 ± 8.35c	5.12 ± 0.27a	1.87 ± 0.28bc	52.13 ± 2.65c	153.28 ± 19.84b	2.93 ± 0.32b
SY24	204.72 ± 5.56d	5.47 ± 0.17a	0.92 ± 0.03c	37.67 ± 1.95d	225.26 ± 12.75a	6.01 ± 0.30a

We then compared soil TC, TN, TP contents, and stoichiometric ratios in different soil layers within the same laying years of straw checkerboard barriers (Fig. 2). We found that in the CK group, soil TC content and C:N ratio in the 0–20 cm soil layer were significantly higher than in the 20–30 cm layer, whereas the soil N:P ratio showed opposite trend and other differences did not show any significance (*P* < 0.01). In the SY1 group, soil TC content (*P* < 0.01), C:P ratio (*P* < 0.01), and C:N ratio (*P* < 0.001) in the 0–20 cm soil layer showed a significant increase compared to the 20–30 cm layer. Moreover, soil TN content and N:P ratio displayed opposite trends (*P* < 0.001), and no significant differences were recorded in soil TP content. In the SY2 group, we only recorded significantly higher soil C:P ratio in the 0–20 cm soil layer compared to the 20–30 cm layer (*P* < 0.05), while no other significant differences were observed. In the SY5 group, significantly higher soil TN content in the 0–20 cm soil layer was observed compared to the 20–30 cm layer (*P* < 0.05). Moreover, we observed a significantly opposite trend in soil C:N ratio (*P* < 0.05) and C:P ratio (*P* < 0.01), while no other significant differences were observed. In the SY10 group, significantly higher soil TC content (*P* < 0.05), N:P ratio (*P* < 0.05), and C:P ratio (*P* < 0.01) in the 0–20 cm soil layer were observed compared to the 20–30 cm layer, while no other significant differences were recorded. In the SY24 group, significantly higher soil TP content in the 0–20 cm soil layer was observed compared to the 20–30 cm layer. Whereas, the soil C:P ratio displayed an opposite trend and no other significant differences were recorded (*P* < 0.01).

### Changes in soil contents of MBC, MBP, and MBN and stoichiometric ratios in straw checkerboard barriers across different laying years

**Figure 3:**
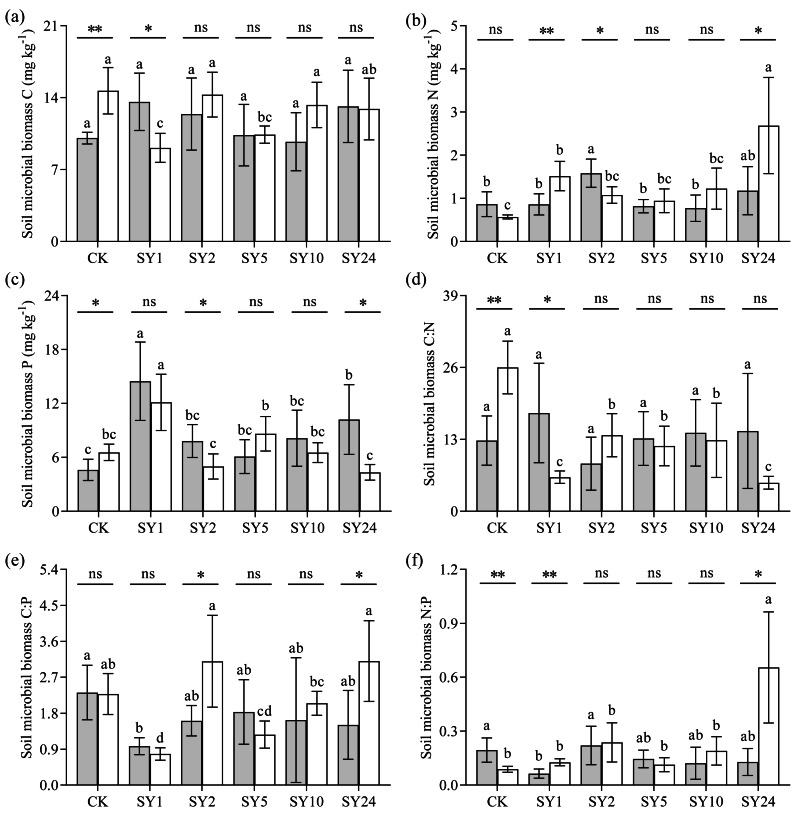
(A–F) Changes in soil contents of MBC, MBP, and MBN and stoichiometric ratios. CK represents a bare sandy area without straw checkerboard barriers, while SY1, SY2, SY5, SY10, and SY24 represent straw checkerboard barriers of different laying years. Lowercase letters (such as a, b, c, d, ab, bc, and cd) denote significant differences between laying years of straw checkerboard barriers (*P* < 0.05). Significant variations (such as *** , ** , * , ns) between different soil layers (0–20 cm, 20–30 cm) of straw checkerboard barriers with the same laying year are indicated by asterisks ** (*P* < 0.01), * (*P* < 0.05), ns (no significant difference). Values are mean s ± S D (*n* = 5).

We compared soil contents of MBC, MBP, and MBN and stoichiometric ratios in straw checkerboard barriers across different laying years (Fig. 3). We found no obvious regular variations in the soil contents of MBC, MBP, and MBN and MBC:MBP, MBN:MBP, and MBC:MBN ratios in the 0–20 cm and 20–30 cm soil layers across different laying years of straw checkerboard barriers.

We then compared soil MBC, MBP, and MBN contents and stoichiometric ratios in different soil layers within the same laying years of straw checkerboard barriers (Fig. 3). In the CK group, we found significantly higher soil MBN:MBP ratio in the 0–20 cm soil layer compared to the 20–30 cm layer (*P* < 0.01), while soil contents of MBC (*P* < 0.01) and MBP (*P* < 0.05), and MBC:MBN ratio (*P* < 0.01) displayed opposite trend. No significant differences were observed in soil MBN content and MBC:MBP ratio. In the SY1 group, significantly higher soil MBC content and MBC:MBN ratio in the 0–20 cm soil layer were observed compared to the 20–30 cm layer (*P* < 0.05). Moreover, soil MBN content and MBN:MBP ratio showed an opposite trend (*P* < 0.01), and no significant differences in soil MBP content and MBC:MBP ratios were recorded. In the SY2 group, we recorded significantly higher soil MBP and MBN contents in the 0–20 cm soil layer compared to the 20–30 cm layer, while the soil MBC:MBP ratio displayed an opposite trend (*P* < 0.05). No other significant differences were recorded in this group. In the SY5 and SY10 groups, we did not observe significant differences in soil contents of MBC, MBP, and MBN and ratios of MBC:MBN, MBN:MBP, and MBC:MBP in the 0–20 cm and 20–30 cm soil layers. In the SY24 group, significantly higher soil MBP content in the 0–20 cm soil layer was recorded compared to the 20–30 cm layer, while soil MBN content and ratios of MBN:MBP and MBC:MBP displayed opposite trends (*P* < 0.05). We did not observe significant differences in soil MBC content and MBC:MBN ratio.

### Regression analysis of C, P, N contents and stoichiometric ratios of straw, soil, and soil microbial biomass across different laying years of straw checkerboard barriers

**Figure 4:**
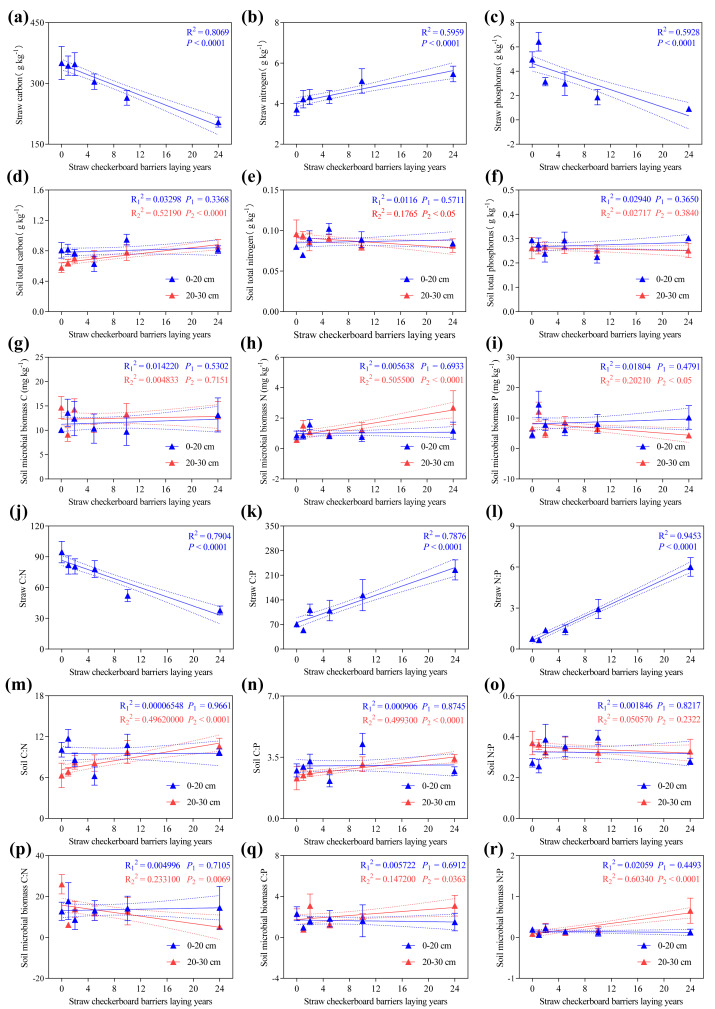
Regression analysis of contents of C, P, and N and stoichiometric ratios of straw, soil, and soil MB. (A) Straw carbon; (B) straw nitrogen; (C) straw phosphorus; (D) soil total carbon; (E) soil total nitrogen; (F) soil total phosphorus; (G) soil MBC; (H) soil MBN; (I) soil MBP; (J) straw C:N; (K) straw C:P; (L) straw N:P; (M) soil C:N; (N) soil C:P; (O) soil N:P; (P) soil MB C:N; (Q) soil MB C:P; (R) soil MB N:P.

We recorded a significantly negative correlation between straw C and P contents, straw C:N ratio with different laying years of straw checkerboard barriers (*P* < 0.0001) (Fig. 4). A significantly positive correlation between straw N content, straw C:P, and straw N:P ratios with different laying years of straw checkerboard barriers (*P* < 0.0001) was observed. The stoichiometric ratios, soil contents of C, P, and N, and soil MB in the 0–20 cm soil layer showed a relatively weak correlation with different laying years of straw checkerboard barriers. The contents of TC and MBN, ratios of C:N and C:P , and MBN:MBP ratio of soil in the 20–30 cm soil layer displayed a significantly positive correlation with different laying years of straw checkerboard barriers (*P* < 0.0001). Moreover, soil contents of TN and MBP displayed a strong negative correlation with different laying years of straw checkerboard barriers (*P* < 0.05).

### Correlations analysis of contents of C, P, and N and stoichiometric ratios of straw, soil, and soil microbial biomass with different laying years in straw checkerboard barriers

**Figure 5:**
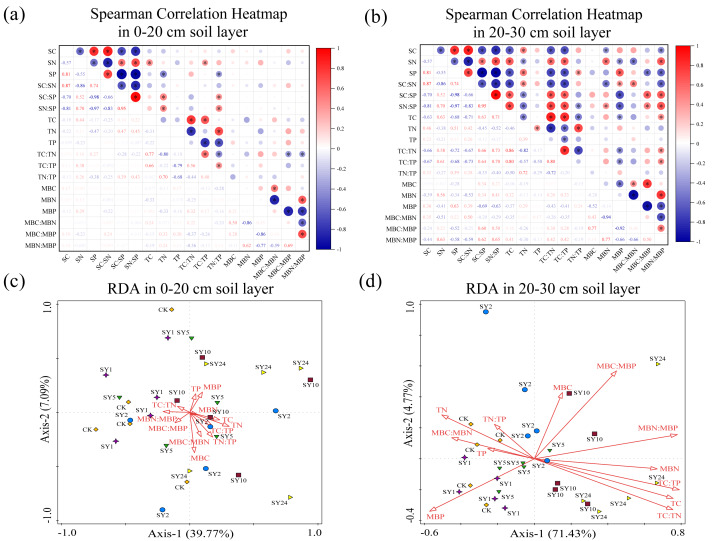
Redundancy analysis (RDA) (A, B) and Spearman correlation heatmap (C, D) among contents of C, P, and N and stoichiometric ratios of straw, soil, and soil MB (* *P* < 0.05). (SC) straw carbon; (SN) straw nitrogen; (SP) straw phosphorus; (TC) soil total carbon; (TN) soil total nitrogen; (TP) soil total phosphorus; (MBC) soil microbial biomass C; (MBN) soil microbial biomass N; (MBP) soil microbial biomass P.

The correlation and RDA analysis (Fig. 5) indicated a definite link among the contents of C, P, and N and stoichiometric ratios of straw, soil, and soil MB in straw checkerboard barriers across different laying years. A significantly positive correlation of the straw N content with soil TC content and soil C:P ratio (*P* < 0.05) was recorded in the 0–20 cm soil layer (Fig. 5A). The straw P content displayed a substantial negative correlation with soil TN content and soil N:P ratio (*P* < 0.05). A significantly positive correlation of straw C:P and straw N:P ratios with soil TN and soil N:P ratio (*P* < 0.05) was found. The soil C:N ratio displayed a distinctly negative correlation with soil MBN:MBP and MBC:MBP ratios (*P* < 0.05). A significantly negative correlation of straw C content with soil contents of TC and MBN, soil C:P, C:N, and MBN:MBP ratios were observed in the 20–30 cm soil layer (Fig. 5B). On the other hand, a considerably positive correlation with soil TN content (*P* < 0.05) was recorded. The straw N content showed a significantly negative correlation with soil contents of TN and MBP, and soil MBC:MBN ratio, while substantially positive correlation with contents of soil TC and MBN, soil C:P, C:N, and MBN:MBP ratios (*P* < 0.05) was observed. The straw P content showed a considerably negative correlation with soil TC content, soil C:P, C:N, MBN:MBP, and MBC:MBP ratios, whereas a significantly positive correlation with soil contents of TN and MBP and soil N:P ratio (*P* < 0.05) was found. Soil TC content, soil C:P, and C:N ratios showed a significant negative and positive correlation with soil MBP content and soil MBN:MBP ratio (*P* < 0.05), respectively. The total cumulative explanatory degrees of C, P, and N contents and stoichiometric ratios of soil and soil MB in 0–20 cm and 20–30 cm soil layers were 46.86% and 76.20%, respectively (Figs. 5C, 5D). The Monte Carlo test was used to further evaluate the factors influencing the changes in straw C, P, and N contents (Table 2). According to the results, the order of the influence of each factor in the 0–20 cm soil layer was MBN:MBP>TC>MBP>TC:TN>MBN>TN>TC:TP>MBC:MBN>TP>MBC>MBC:MBP> TN:TP. However, the order in which each component affected the 20–30 cm soil layer was TC:TP>MBN:MBP>TC>MBC:MBP>MBC:MBN>TC:TN>MBP>MBC>MBN>TN>TP> TN:TP. This explained variation reached a significant value of 44.8% and 13.7% for TC:TP and MBN:MBP, respectively (*P* < 0.01).

**Table 2:** Monte Carlo test results for 0–20 cm and 20–30 cm soil layers (**, *P* < 0.01).

0–20 cm soil layer				20–30 cm soil layer		
Environmental factor	Variance explained (%)	Significance *P*		Environmental factor	Variance explained (%)	Significance *P*
MBN:MBP	7.6	0.126		TC:TP	44.8	0.002**
TC	6.3	0.168		MBN:MBP	13.7	0.006**
MBP	5.1	0.196		TC	3.5	0.078
TC:TN	4.5	0.262		MBC:MBP	3.3	0.122
MBN	4.0	0.268		MBC:MBN	2.5	0.190
TN	3.9	0.300		TC:TN	2.4	0.208
TC:TP	3.9	0.232		MBP	1.8	0.268
MBC:MBN	3.3	0.286		MBC	1.5	0.350
TP	3.1	0.318		MBN	1.5	0.352
MBC	3.1	0.382		TN	0.9	0.490
MBC:MBP	2.2	0.414		TP	0.6	0.622
TN:TP	0.3	0.858		TN:TP	0.1	0.936

**Figure 6:**
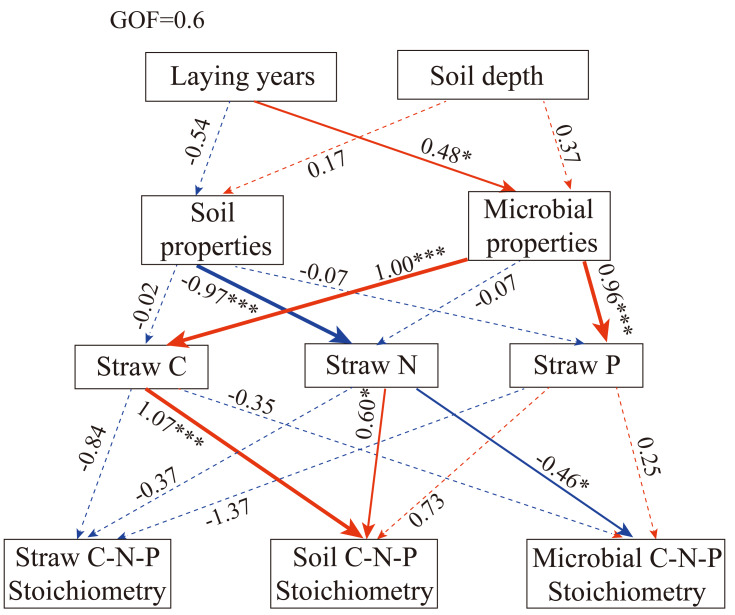
Structural equation model (SEM) showing the influence of laying years, soil depth, and contents of C, N, and P on straw-soil-microbial C-N-P stoichiometry and soil and microorganisms properties. All variables were categorized into groups to make the study easier; (i) laying years comprised of 1, 2, 5, 10, and 24 years, (ii) soil depth consists of 0–20 cm and 20-30 cm soil layers, (iii) soil properties include soil TC, TP, and TN, (iv) microbial properties include soil MBC, MBP, and MBN, (v) straw C-N-P stoichiometry show s straw C:N, N:P, and C:P ratios (vi) soil C-N-P stoichiometry displays soil C:N, N:P, and C:P, ratios, (vii) microbial C-N-P stoichiometry represents soil MBC:MBN, MBN:MBP, and MBC:MBP ratios. The width of the arrow corresponds to the degree of path coefficients, and adjoining integers marked in the same direction as the arrow represent path coefficients. Positive and negative associations are denoted by blue dashed lines and red solid lines, respectively. Different levels of significance are represented with * *P* < 0.05, and *** *P* < 0.001. Goodness-of-fit (GOF) index is equal to 0.6.

The SEM results (Fig. 6) revealed a standardized direct negative effect of straw contents of C (−0.84), N (−0.37), and P (−1.37) on the C-N-P stoichiometric ratio of straw. The straw contents of C (1.07), N (0.60), and P (0.73) displayed standardized direct positive effects on the C-N-P stoichiometric ratio of soil, whereas straw contents of C (*P* < 0.001) and N (*P* < 0.05) had standardized directly and significantly positive effects. The straw content of C (−0.35) and N (−0.46) directly and negatively influenced the C-N-P stoichiometric ratio of soil MB, while straw P content (0.25) directly and positively affected the C-N-P stoichiometric ratio of soil MB, and straw N content had standardized directly and significantly negative effects (*P* < 0.05). The soil and microbial properties showed variations in the straw contents of C, P, and N as the laying years of straw checkerboard barriers and soil depth increased which subsequently influenced the stoichiometric ratios of straw, soil, and soil MB. Among them, microbial properties primarily influenced straw C and P contents which in turn indirectly affected the stoichiometric ratios of soil and soil MB. Similarly, soil properties firstly impacted straw N content, and subsequently influenced stoichiometric ratios of straw and soil MB.

## Discussion

The plant straw of straw checkerboard barriers undergoes decomposition to varied degrees as the number of laying years increases. The C, N, and P elements in the straw are gradually transformed by microbes into different forms such as humus, ammonium nitrogen, and inorganic phosphate, which are then absorbed and utilized by the plants (Qu et al., 2006; Mao et al., 2022). The results of this study indicated that as the laying years of straw checkerboard barriers increased, straw C content decreased, straw N content increased, while straw P content initially increased and then decreased. Previous studies have found that the C element in the straw is utilized and transformed by microbes into carbon dioxide and other forms, partially releasing it into the atmosphere, leading to a gradual decrease in straw C content, which is consistent with the results of this study (Han et al., 2024). The stoichiometric ratio of straw influences the composition and abundance of soil microorganisms, which in turn impacts functions of the soil ecosystem such as nutrient cycling and organic matter decomposition (Trisha et al., 2021; Dzyuin, 2018). This study found that as the laying years of straw checkerboard barriers increased, the straw C:N ratio gradually decreased, while straw N:P and C:P ratios gradually increased. Sun et al. (2021) reported that C, N, and P were gradually released into the soil as straw buried in soil decomposed. The C was released in higher quantities than P, while N was released in slightly larger quantities than P. Similar to the findings of this study, straw N:P and C:P ratios gradually increased, enhancing soil nutrient availability.

The decomposition of straw in sandy soil releases nutrients such as C, N, and P, increasing their storage in both surface and deeper soil layers. This process enhances soil fertility, influences the soil stoichiometric ratio, and promotes nutrient cycling and energy flow within the soil ecosystem (Wang et al., 2023; Trisha et al., 2021). By comparing the contents of soil total carbon (TC), total phosphorus (TP), and total nitrogen (TN), and stoichiometric ratios of different laying years of straw checkerboard barriers, we found that soil contents of TC and TN showed irregular changes in the 0–20 cm soil layer as the laying years of straw checkerboard barriers increased. One possible explanation could be that the addition of sandy soil and straw layers impacted the microbial decomposition, leading to altered microbial activities. Mu et al. (2011) and Liu et al. (2023) reported that compared to the absence of straw incorporation, returning straw to the field significantly increased the soil content of TN in the 0–20 cm soil layer, and also enhanced the C storage across all soil layers by 8% to 13%. The N and P reserves in the surface soil also increased significantly by 9% and 5%, respectively, which differs from the findings of this study (Mu et al., 2011; Liu et al., 2023). Soil stoichiometric ratio indicates the state of nutrients that the soil provides to plants, influences the growth and metabolism of soil microorganisms, and varies in its optimal range depending on different plant species (Zhang et al., 2013; Xu et al., 2017). Studies have shown that straw contains a significant amount of C, which can increase soil C:P and C:N ratios, leading to a more balanced nutrient supply and providing a more suitable environment for soil microorganisms (Cong et al., 2020; Zhang et al., 2017). This study found that soil C:P and C:N ratios increased in the 20–30 cm soil layer as the laying years of straw checkerboard barriers increased, consistent with previous findings.

Straw contains abundant nutrients such as C, N, P, and organic matter, serving as an essential energy and nutrient source for soil microorganisms, thereby stimulating microbial proliferation and increasing both their population and biomass (Cui et al., 2014). Straw decomposition rate is strongly influenced by soil microbial biomass, levels of activity, and structure of the community and different microorganisms exhibit different abilities to break down different straw components (Yu et al., 2023). This study found that soil contents of MBC, MBP, and MBN initially increased and then decreased in a short period of laying years, even though they did not show any significant changing pattern in the 0–20 cm and 20–30 cm soil layers as the laying years of straw checkerboard barriers increased. Previous studies have shown that returning straw to the field significantly enhanced the soil contents of MBC, MBP, and MBN, while other studies suggested that some soil N might be fixed during the straw decomposition, leading to a temporary decrease in soil MBN content. However, soil MBN content gradually increases as the progression of straw decomposition releases N. This differs from the findings of this study, possibly due to variations in straw species, soil types, and returning methods (Guo et al., 2015; Chen et al., 2013; Zhang, Ren & Cai, 2024).

During straw decomposition, soil microorganisms utilize and transform nutrients such as C, N, and P according to their growth requirements, thereby exerting a feedback regulatory effect on the soil stoichiometric ratio (Nie et al., 2021; Song et al., 2024). Microorganism activity and diversity can be increased with the use of an appropriate soil stoichiometric ratio (Hu et al., 2023; Pan et al., 2022). Straw checkerboard barriers laying years, straw, soil, and soil MBC, MBP, and MBN, and their stoichiometric ratios are all intricately and closely related. This study found that Straw N and soil MBN contents were shown to be considerably favorably connected with the number of years spent constructing straw checkerboard barriers, while soil TN content was found to be significantly negatively correlated. This suggests that as the laying years of straw checkerboard barriers increased, soil microorganisms released less N from straw while absorbing and transforming soil TN content was relatively high. This study also found that in the later stage of straw checkerboard barrier laying, straw decomposition accelerated, leading to a substantial release of C, which temporarily increased soil C:N ratio over a certain period (Zhou et al., 2024; Liu et al., 2024). This finding is inconsistent with previous research findings, possibly due to the initially slow microbial decomposition rate of straw in sandy soil and fewer microorganisms capable of decomposing C elements were present. Microorganisms absorb and utilize P and N in a certain proportion, causing a gradual change in the soil N:P ratio during the ecological restoration process after laying straw checkerboard barriers. When the N supply in the soil is relatively sufficient, it will lead to an increase in soil N:P ratio (Sun et al., 2023; Wang et al., 2022). The SEM results revealed that straw C and N contents had a direct and significantly positive effect on the soil C-N-P stoichiometric ratio, whereas the straw N had a direct and significantly negative effect on the soil MB C-N-P stoichiometric ratio. The C-N-P stoichiometric ratio reflects the cycling rate and coupling dynamics of C, N, and P elements in the ecosystem. Changes in the straw contents of C, P, and N affect the C, P, and N contents of soil and soil MB (Sun et al., 2022; Wang & Yu, 2008).

This study has, to some extent, revealed the dynamic balance laws of C, N, P in the ecosystem of straw checkerboard barriers across different laying years. In the future, the changes in C, N, and P in straw, soil, and soil MB across different laying years of straw checkerboard barriers in different regions and under different climatic conditions can be simulated and predicted by developing an ecosystem model based on multiple parameters and integrating remote sensing technology. This will provide scientific decision support for desertification prevention and control projects.

## Conclusions

The laying years of straw checkerboard barriers exert varying effects on the C, N, P stoichiometric characteristics of straw, soil (0–20 cm and 20–30 cm layers) and soil microorganisms.For straw, longer laying years increased N content, N:P and C:P ratios, decreased C content and C:N ratio, and led to a first increase then decrease in P content. For soil, the 0–20 cm layer had no significant stoichiometric changes with laying years; the 20–30 cm layer showed increased TC content, C:P and C:N ratios, a first increase then decrease in TP content, while TN content and N:P ratio did not show any significant changes. Soil microbial biomass (MBC, MBN, MBP) in both the 0–20 cm and 20–30 cm soil layers showed no overall significant correlation with laying years, but fluctuated first increasing then decreasing in the early stage. Regression analysis showed correlations between laying years and straw stoichiometry; straw N contents affected soil stoichiometry positively, while straw C and P content inhibited microbial biomass stoichiometry. The main factors controlling straw C, N, and P contents were soil MBN:MBP ratio and TC content in the 0–20 cm layer, and soil TC:TP and MBN:MBP ratios in the 20–30 cm layer.

##  Supplemental Information

10.7717/peerj.21569/supp-1 Supplemental Information 1 The coefficient of variation (CV, %) in straw C, P, N contents and stoichiometric ratios in straw checkerboard barriers across different laying years (mean ± SE) Note: CK represents bare sandy area without straw checkerboard barriers, while SY1, SY2, SY5, SY10 and SY24 represent straw checkerboard barriers of different laying years.

10.7717/peerj.21569/supp-2 Supplemental Information 2 Raw data

## Additional Information and Declarations

### Data Availability

The following information was supplied regarding data availability: The raw data is available in the Supplemental Files.
