# Supplementary material for: Ecological stoichiometric characteristics of the straw-soil-microbial system in the straw checkerboard barriers across different laying years
Source: PeerJ. 2026 Jul 30;14:e21569. doi: 10.7717/peerj.21569 (PMC13429105; doi:10.7717/peerj.21569)
Supplement: Supplemental Information 1 — Note: CK represents bare sandy area without straw checkerboard barriers, while SY1, SY2, SY5, SY10 and SY24 represent straw checkerboard barriers of different laying years. [file peerj-14-21569-s001.docx]

| Research plots | Straw carbon (%) | Straw nitrogen (%) | Straw phosphorus (%) | Straw C:N (%) | Straw C:P (%) | Straw N:P (%) |
| --- | --- | --- | --- | --- | --- | --- |
| CK | 11.63 | 8.20 | 6.38 | 11.10 | 7.72 | 6.62 |
| SY1 | 5.65 | 10.17 | 11.93 | 10.81 | 11.58 | 13.93 |
| SY2 | 8.15 | 8.70 | 10.94 | 9.14 | 14.80 | 13.64 |
| SY5 | 6.23 | 6.96 | 32.51 | 10.58 | 26.67 | 25.89 |
| SY10 | 7.06 | 11.84 | 33.68 | 11.38 | 28.94 | 24.15 |
| SY24 | 6.07 | 7.03 | 7.97 | 11.58 | 12.65 | 11.30 |
